# Transcranial brain stimulation: Past and future

**DOI:** 10.1177/2398212818818070

**Published:** 2018-12-04

**Authors:** John Rothwell

**Affiliations:** UCL Queen Square Institute of Neurology, University College London, London, UK

**Keywords:** Transcranial magnetic stimulation, transcranial direct current stimulation, virtual lesion, synaptic plasticity

## Abstract

This article provides a brief summary of the history of transcranial methods for stimulating the human brain in conscious volunteers and reviews the methodology and physiology of transcranial magnetic stimulation and transcranial direct current stimulation. The former stimulates neural axons and generates action potentials and synaptic activity, whereas the latter polarises the membrane potential of neurones and changes their sensitivity to ongoing synaptic inputs. When coupled with brain imaging methods such as functional magnetic resonance imaging or electroencephalography, transcranial magnetic stimulation can be used to chart connectivity within the brain. In addition, because it induces artificial patterns of activity that interfere with ongoing information processing within a cortical area, it is frequently used in cognitive psychology to produce a short-lasting ‘virtual lesion’. Both transcranial magnetic stimulation and transcranial direct current stimulation can produce short-lasting changes in synaptic excitability and associated changes in behaviour that are presently the source of much research for their therapeutic potential.

## Early beginnings

The first modern experiments on brain stimulation were carried out on dogs by Fritz and Hitzig in Germany and on primates by Ferrier in the United Kingdom. The experimenters removed the skull and showed that electrical stimulation of central areas of cortex produced movements of the opposite side of the body. It was only a few years after these observations in animals that an American surgeon, Roberts Bartholow, used faradic stimulation (a form of alternating current) for the first time on the human brain. In 1874, he stimulated the exposed central region of the cerebral cortex in a patient whose scalp had been eroded by a tumour and produced movements and localised sensations of the opposite side of the body (see details in Harris and Almerigi (2009)). In the early 20th century, neurosurgeons began to use localised cortical stimulation more routinely to localise ‘eloquent’ areas of the brain that they might wish to avoid during surgical procedures.

Neurosurgeons used ‘faradic’ stimulation (usually 60 Hz (United States) or 50 Hz (Europe) alternating current) applied for 0.5 s to several seconds to examine cortical responsiveness in the exposed brain of conscious patients. However, attempts to use similar methods to activate the brain through the intact scalp often failed (see Merton (1981); with one successful report by Gualtierotti and Paterson (1954)). This was because with two electrodes attached to the surface of the scalp, most of the applied current flows through the skin between the electrodes. Only a small fraction penetrates the high resistance of the skull and scalp. The result is that when there is enough current in the brain to activate neurones, there is a much larger current along the scalp, which is painful because of the activation of local nerve endings as well electrically induced contraction of scalp muscles. The effect is compounded by the duration of the applied stimulus, and effectively, brain stimulation remained in the purview of neurosurgeons.

## The last 50 years

In 1980, Merton and Morton revisited the problem of transcranial brain stimulation. They used a stimulator that they had developed to activate directly muscle fibres through the skin (rather than via the muscle nerve). The stimulator worked with a very-high voltage that they thought would reduce the resistance of the skin under the stimulating electrodes and allow current to penetrate the brain more readily. Although this is probably not the case, they were nevertheless successful and found that stimulation of motor cortex with just one high-intensity pulse was sufficient to elicit movement of the opposite side of the body. The concept of transcranial brain stimulation was born.

The advantage of the method over previous attempts was the simple fact that a single stimulus is much less painful than a series of stimuli continuing for several seconds. Even so, it still generates a considerable contraction of scalp muscles, which is usually perceived as uncomfortable even if it is far more acceptable than 50 Hz stimulation for several seconds. Although the method was used for several years, it was only with the development of transcranial magnetic stimulation (TMS) (Barker et al., 1985) that a comfortable and acceptable method became available for non-invasive transcranial stimulation of the human brain (see below).

Following the development of TMS, a number of other older approaches were re-tested for their effectiveness on activation of human brain. The most commonly used is transcranial direct current stimulation (tDCS), which involves applying a small (1–2 mA), continuous current between scalp electrodes for periods of from several seconds to many minutes (Nitsche and Paulus, 2000) (see below). From the preceding discussion about scalp resistance, it is clear that tDCS can only produce very small electrical currents in the brain, which are insufficient to generate action potentials in neurones. Instead it ‘polarises’ neurones, which means that it changes their transmembrane electrical potential by 0.5–1 mV. The effect is to make the neurones slightly more or less difficult to activate by ongoing activity in the brain. This is sometimes referred to as ‘neuromodulation’. The advantage of tDCS is that the equipment is far simpler to make than for TMS, and the price is correspondingly lower.

## TMS

TMS employs the principle of electromagnetic induction, first discovered by Michael Faraday, in which a changing magnetic field gives rise to a companion electric field which induces electric currents in nearby conductive structures. In the case of TMS, a large pulse of current in the external stimulating coil generates a rapidly changing magnetic field that rises to, and falls from, 1 Tesla or more within 1 ms. This field can penetrate the scalp and skull with little impedance, and the electrical field it induces causes currents to flow in the brain. These are of the same order as those used in conventional electrical stimulation of the exposed brain and excite axons of neural elements (Barker et al., 1991; Peterchev et al., 2008), mainly in superficial structures within the cerebral cortex. TMS is more acceptable than the Merton and Morton’s method because the electric currents induced in the scalp are much smaller and similar in magnitude to those induced in the brain itself.

The design of the external stimulating coil affects the distribution of induced field in the brain. A simple circular coil (usually 7–10 cm in diameter) induces currents that are maximal in an annulus under the coil. A figure-of-eight coil, which consists of two overlapping circular coils, is more focal since the induced currents under the intersection of the circles are twice as strong as those at the periphery (Ueno and Matsuda, 1992). The area of stimulation depends on the diameters of the two coils and the intensity of stimulation, but as a rule of thumb, the commonly used double 8-cm coil stimulates an area of approximately 2–4 sq. cm.

The outcome is that TMS provides a simple and easily used way of directly stimulating and inducing action potentials, in cerebral neurones. The limitations are focality and stimulation depth. A magnetic field cannot be focussed in the way a lens can focus light, so the method is limited to activating relatively large volumes of tissue compared with a conventional surface electrode. Depth of stimulation is limited by the design of the coil. With conventional coils, stimulus falls off rapidly with distance from the coil, being only about 30% as effective at 5 cm compared with the coil surface. Given the distance across the skull from the coil to the brain, combined with the depth of human cortical sulci at around 2 cm, these coils can be imagined to stimulate all parts of the cerebral cortex that would be visible if the skull were completely removed. Different designs of coil can generate more stimulation at depth, but at the cost of focality and always with the consequence that superficial structures will be stimulated more powerfully than those at depth.

## tDCS

Early forms of tDCS were investigated in the 1960s and 1970s. They emerged from animal work that had shown the DC polarisation of the exposed cortex in animals could increase or decrease the ongoing activity that could be recorded (Bindman et al., 1962). In addition, it had been found that several minutes of polarisation could lead to lasting effects on excitability, with discharge rates being increased or decreased for hours or more following 10-min polarisation. In human brain, tDCS involves passing a 1- to 2-mA constant current between electrodes secured to the scalp. Conventionally, the current flows from the positive electrode (anode) to the negative electrode (cathode). If the cortical surface was smooth (lissencephalic, that is, without sulci), then neurones, such as pyramidal neurones, that are oriented perpendicularly to the scalp surface would be depolarised at their cell bodies if they were located under the surface anode. This might make them slightly easier to discharge by any ongoing synaptic inputs (explaining the increase in discharge seen in the animal experiments), and conversely reducing excitability of neurones under the cathode. However, the real situation is likely to be much more complex, and modelling the effects of tDCS on a folded cortex is currently under development.

## TMS and tDCS: the story so far

TMS and tDCS are remarkable tools that greatly expand our ability to interact with processing in the central human nervous system. Not only can we view in great detail (magnetic resonance imaging (MRI), electroencephalography (EEG), etc.) the anatomy and activity in the brain, we can now stimulate neurones in brain circuits and directly affect ongoing activity. Some of the common uses are described below.

### TMS and connectivity

When the motor cortex is stimulated, we can observe a contraction of contralateral muscles. We can, therefore, test the connection between cortex and muscle, for example, for its conduction velocity or its excitability. The existence of a connection is a confirmation of an anatomical pathway, whereas the properties of the connection can give us information about how well the pathway operates. For example, in multiple sclerosis, the pathway from cortex to muscle is much slower than normal because of central demyelination (Hess et al., 1986). The concept can be expanded to connections between areas of cortex. For example, there are connections from motor cortex to other parts of the brain as well as to muscles. These other connections can be observed with other techniques such as functional magnetic resonance imaging (fMRI) (TMS-fMRI) or EEG (TMS-EEG). Thus, TMS of motor cortex can be seen to activate connections to basal ganglia, thalamus, cerebellum and many other areas (Bestmann et al., 2004). In fact, the excitability of a particular connection depends on its excitability at the time the TMS is given. This means that if a pathway between (e.g. frontal eye fields and visual cortex) is active in a task, then the strength of the connection appears to increase when tested with TMS (Ruff et al., 2006). Connectivity also changes with levels of consciousness: stimulation of a cortical area usually is followed by spread of activity to very many distant cortical areas in alert volunteers, but is much reduced in sleep or in disorders of consciousness (Massimini et al., 2009). Indeed, it has been suggested that quantifying the complexity of spread of neural activity could be an objective marker of brain state in the unconscious patient (Ragazzoni et al., 2013). Finally, some relatively direct connections are known to employ particular neurotransmitters, such as GABA, and these are regularly used to measure excitability of GABAergic connections in brain (Kujirai et al., 1993; Ziemann et al., 2015).

### TMS and ‘virtual lesions’

Although TMS produces action potential in neurones, it will also disrupt any ongoing patterns of activity that were present before the stimulus (Walsh and Cowey, 2000). The outcome is that if an area is actively contributing to a behaviour when TMS is applied, then the behaviour is disturbed. For example, if a visual stimulus is briefly presented on a screen for 1–2 ms, then the primary visual cortex actively processes that data 60+ ms later (Maccabee et al., 1991). If a TMS pulse is applied at around that time, perception of the stimulus is reduced and participants may be unable to see the stimulus even though it is clear to other people in the room. This effect is sometimes termed a ‘virtual lesion’ and has been used extensively in cognitive neuroscience to test whether activity in an area is necessary for task performance. A classic study asked whether activity that could be seen in visual cortex with fMRI when congenitally blind people read Braille letters was helping them perform the task, even though no visual input was being processed (Cohen et al., 1997). They found that TMS to the visual areas disrupted blind Braille reading even though it had no effect on sighted volunteers who read embossed Roman letters. The conclusion was that the visual cortex activity was somehow contributing to the ability of these blind people to read Braille.

### TMS, tDCS and ‘plasticity’

This is the topic which is generating most interest at present. Many experiments have shown that repetitive TMS (rTMS) with several hundred stimuli given over a short period of time, or 10 min or more of tDCS can both lead to effects on the cortex that outlast the period of stimulation by many minutes or hours (Ziemann et al., 2008). The hypothesis is that these forms of stimulation can interact with synaptic plasticity, increasing or decreasing the excitability of neural connections in the cortex in a manner similar to long-term potentiation/depression (LTP/LTD) in animal experiments. The idea is exciting because if it is possible to produce long-term changes in synaptic function, then there is the possibility of using these methods therapeutically in neurological disease.

For rTMS, the hypothesis is that each stimulus pulse activates the same set of synaptic connections. By analogy with work in the hippocampus, repeated activation of synaptic connections may lead to long-term changes in the effectiveness of the connection, hence an effect on synaptic plasticity. For tDCS, the mechanism is less clear. Since tDCS cannot directly discharge neurones, its effects must depend on the ongoing activity in system during the period of tDCS. In animal experiments, this has been shown to cause brain derived neurotrophic factor (BDNF)-dependent increases in synaptic efficacy (Fritsch et al., 2010).

So far, this approach has led to the introduction of rTMS as a therapy for treatment-refractory depression (O’Reardon et al., 2007). Current trials are also underway in many other conditions including rehabilitation after stroke and treatment of tinnitus and neuropathic pain.

## Brain stimulation: the future

### Improving present methodologies

Apart from the success in treating depression, rTMS and tDCS have produced more variable and less-effective results in other conditions. There are many possible reasons for this, including the suitability of the condition itself, but most attention now is focussed on improving the methodologies to reduce the variation in response both between individuals and on the same individual from day to day (Ridding and Ziemann, 2010). Two factors may be relevant: first, the effects of any brain stimulation method depend on the brain state at the time the stimulus is applied. Controlling the brain state (e.g. by some focussed behavioural task) or applying stimulation only during a particular brain state, as identified, for example, by patterns of EEG activity, may be one approach that will improve responsiveness (Goldsworthy et al., 2016). A second factor is that both TMS and tDCS activate many different types of neurones, which may be inhibitory or excitatory, or interneurones versus projection neurones. Methods to make TMS more selective involve examining changes in the pulse waveform of stimulation to match the best form to activate particular types of neurone and increasing the focality of tDCS by stimulating through multiple electrodes in order to achieve a more focal field in the brain (D’Ostilio et al., 2016).

### Extending present methodologies

tDCS specifically applies a constant current to the brain in order to produce a sustained polarisation of neural membranes. A relatively new version of this method is transcranial random noise stimulation (tRNS), which applies a 1- to 2-mA alternating current at frequencies similar to those seen in ongoing EEG (e.g. 10 Hz, equating to the EEG alpha rhythm or 20 Hz equating to the beta rhythm). Reduced animal preparations have shown that such currents are capable of entraining oscillating activity in neural populations at the applied frequency. It is thought that the same may occur in the human brain (Ali et al., 2013). Such entrainment of brain activity has been shown to modulate or even suppress ongoing tremor activity in patients with Parkinson’s disease (Brittain et al., 2013). Other studies have examined the role of frequency coupling in governing the interaction of distant areas of brain in cognitive tasks. For example, EEG activity in the parietal and frontal areas of cortex appears to be briefly synchronised at around 6 Hz when volunteers perform a working memory task. Artificial synchronisation of activity at this frequency in these two areas with tACS can improve performance on the task, suggesting that it might be possible to enhance interaction between brain areas (Polania et al., 2012).

A second area of interest is the new technique of focussed pulsed ultrasound (US). US has the advantage over TMS and tDCS in that it can be focussed onto targets deep in the brain. Neurosurgeons have made use of this using focussed US to heat very small volumes of brain to produce permanent lesions. For example, focussed US is now a recognised method for producing small lesions in the thalamus to treat tremors (Elias et al., 2016). If a different frequency of US is applied, there is now good evidence from animal experiments that it can stimulate neurones rather than destroy them (Tufail et al., 2010). The mechanism of the effect is unknown. It could be an effect of the US on the nerve membrane, causing ion channels to open, depolarise the nerve and produce action potentials. Alternatively, the sound could cause fusion of synaptic vesicles with the membrane and release neurotransmitter into synapses. If safety concerns can be satisfied, it may, therefore, become possible in the future to use focussed US to directly activate regions deep in the brain that are currently inaccessible to TMS and tDCS.

## References

[bibr1-2398212818818070] Ali MM Sellers KK Frohlich F (2013) Transcranial alternating current stimulation modulates large-scale cortical network activity by network resonance. Journal of Neuroscience 33(27): 11262–11275. 23825429 10.1523/JNEUROSCI.5867-12.2013 PMC6618612

[bibr2-2398212818818070] Barker AT Garnham CW Freeston IL (1991) Magnetic nerve stimulation: The effect of waveform on efficiency, determination of neural membrane time constants and the measurement of stimulator output. Electroencephalography and Clinical Neurophysiology: Supplement 43: 227–237. 1773760

[bibr3-2398212818818070] Barker AT Jalinous R Freeston IL (1985) Non-invasive magnetic stimulation of human motor cortex [letter]. Lancet 1(8437): 1106–1107. 2860322 10.1016/s0140-6736(85)92413-4

[bibr4-2398212818818070] Bestmann S Baudewig J Siebner HR et al (2004) Functional MRI of the immediate impact of transcranial magnetic stimulation on cortical and subcortical motor circuits. European Journal of Neuroscience 19(7): 1950–1962. 15078569 10.1111/j.1460-9568.2004.03277.x

[bibr5-2398212818818070] Bindman LJ Lippold OC Redfearn JW (1962) Long-lasting changes in the level of the electrical activity of the cerebral cortex produced by polarizing currents. Nature 196(6): 584–585. 13968314 10.1038/196584a0

[bibr6-2398212818818070] Brittain JS Probert-Smith P Aziz TZ et al (2013) Tremor suppression by rhythmic transcranial current stimulation. Current Biology 23(5): 436–440. 23416101 10.1016/j.cub.2013.01.068 PMC3629558

[bibr7-2398212818818070] Cohen LG Celnik P Pascual Leone A et al (1997) Functional relevance of cross-modal plasticity in blind humans. Nature 389(6647): 180–183. 9296495 10.1038/38278

[bibr8-2398212818818070] D’Ostilio K Goetz SM Hannah R et al (2016) Effect of coil orientation on strength-duration time constant and I-wave activation with controllable pulse parameter transcranial magnetic stimulation. Clinical Neurophysiology 127(1): 675–683. 26077634 10.1016/j.clinph.2015.05.017 PMC4727502

[bibr9-2398212818818070] Elias WJ Lipsman N Ondo WG et al (2016) A randomized trial of focused ultrasound thalamotomy for essential tremor. The New England Journal of Medicine 375(8): 730–739. 27557301 10.1056/NEJMoa1600159

[bibr10-2398212818818070] Fritsch B Reis J Martinowich K et al (2010) Direct current stimulation promotes BDNF-dependent synaptic plasticity: Potential implications for motor learning. Neuron 66(2): 198–204. 20434997 10.1016/j.neuron.2010.03.035 PMC2864780

[bibr11-2398212818818070] Goldsworthy MR Vallence AM Yang R et al (2016) Combined transcranial alternating current stimulation and continuous theta burst stimulation: A novel approach for neuroplasticity induction. European Journal of Neuroscience 43(4): 572–579. 26663460 10.1111/ejn.13142

[bibr12-2398212818818070] Gualtierotti T Paterson AS (1954) Electrical stimulation of the unexposed cerebral cortex. The Journal of Physiology 125(2): 278–291. 13192818 10.1113/jphysiol.1954.sp005157 PMC1365819

[bibr13-2398212818818070] Harris LJ Almerigi JB (2009) Probing the human brain with stimulating electrodes: The story of Roberts Bartholow’s (1874) experiment on Mary Rafferty. Brain and Cognition 70(1): 92–115. 19286295 10.1016/j.bandc.2009.01.008

[bibr14-2398212818818070] Hess CW Mills KR Murray NM (1986) Measurement of central motor conduction in multiple sclerosis by magnetic brain stimulation. Lancet 2(8503): 355–358. 2874365 10.1016/s0140-6736(86)90050-4

[bibr15-2398212818818070] Kujirai T Caramia MD Rothwell JC et al (1993) Corticocortical inhibition in human motor cortex. Journal of Physiology 471: 501–519. 8120818 10.1113/jphysiol.1993.sp019912 PMC1143973

[bibr16-2398212818818070] Maccabee PJ Amassian VE Cracco RQ et al (1991) Magnetic coil stimulation of human visual cortex: Studies of perception. Electroencephalography and Clinical Neurophysiology: Supplement 43: 111–120. 1773751

[bibr17-2398212818818070] Massimini M Boly M Casali A et al (2009) A perturbational approach for evaluating the brain’s capacity for consciousness. Progress in Brain Research 177: 201–214. 19818903 10.1016/S0079-6123(09)17714-2

[bibr18-2398212818818070] Merton PA (1981) The first Carmichael memorial lecture: Neurophysiology on man. Journal of Neurology, Neurosurgery, and Psychiatry 44(10): 861–870. 10.1136/jnnp.44.10.861 PMC491170 7031183

[bibr19-2398212818818070] Merton PA Morton HB (1980) Stimulation of the cerebral cortex in the intact human subject. Nature 285(5762): 227. 7374773 10.1038/285227a0

[bibr20-2398212818818070] Nitsche MA Paulus W (2000) Excitability changes induced in the human motor cortex by weak transcranial direct current stimulation. Journal of Physiology 527(Pt. 3): 633–639. 10990547 10.1111/j.1469-7793.2000.t01-1-00633.x PMC2270099

[bibr21-2398212818818070] O’Reardon JP Solvason HB Janicak PG et al (2007) Efficacy and safety of transcranial magnetic stimulation in the acute treatment of major depression: A multisite randomized controlled trial. Biological Psychiatry 62(11): 1208–1216. 17573044 10.1016/j.biopsych.2007.01.018

[bibr22-2398212818818070] Peterchev AV Jalinous R Lisanby SH (2008) A transcranial magnetic stimulator inducing near-rectangular pulses with controllable pulse width (cTMS). IEEE Transactions on Biomedical Engineering 55(1): 257–266. 18232369 10.1109/TBME.2007.900540 PMC3740125

[bibr23-2398212818818070] Polania R Nitsche MA Korman C et al (2012) The importance of timing in segregated theta phase-coupling for cognitive performance. Current Biology 22(14): 1314–1318. 22683259 10.1016/j.cub.2012.05.021

[bibr24-2398212818818070] Ragazzoni A Pirulli C Veniero D et al (2013) Vegetative versus minimally conscious states: A study using TMS-EEG, sensory and event-related potentials. PLoS ONE 8(2): e57069. 23460826 10.1371/journal.pone.0057069 PMC3584112

[bibr25-2398212818818070] Ridding MC Ziemann U (2010) Determinants of the induction of cortical plasticity by non-invasive brain stimulation in healthy subjects. Journal of Physiology 588(Pt. 13): 2291–2304. 20478978 10.1113/jphysiol.2010.190314 PMC2915507

[bibr26-2398212818818070] Ruff CC Blankenburg F Bjoertomt O et al (2006) Concurrent TMS-fMRI and psychophysics reveal frontal influences on human retinotopic visual cortex. Current Biology 16(15): 1479–1488. 16890523 10.1016/j.cub.2006.06.057

[bibr27-2398212818818070] Tufail Y Matyushov A Baldwin N et al (2010) Transcranial pulsed ultrasound stimulates intact brain circuits. Neuron 66(5): 681–694. 20547127 10.1016/j.neuron.2010.05.008

[bibr28-2398212818818070] Ueno S Matsuda T (1992) Magnetic stimulation of the human brain. Annals of the New York Academy of Sciences 649: 366–368. 1580513 10.1111/j.1749-6632.1992.tb49632.x

[bibr29-2398212818818070] Walsh V Cowey A (2000) Transcranial magnetic stimulation and cognitive neuroscience. Nature Reviews Neuroscience 1(1): 73–79. 11252771 10.1038/35036239

[bibr30-2398212818818070] Ziemann U Paulus W Nitsche M et al (2008) Consensus: Motor cortex plasticity protocols. Brain Stimulation 1(3): 164–182. 20633383 10.1016/j.brs.2008.06.006

[bibr31-2398212818818070] Ziemann U Reis J Schwenkreis P et al (2015) TMS and drugs revisited 2014. Clinical Neurophysiology 126(10): 1847–1868. 25534482 10.1016/j.clinph.2014.08.028

